# Retracted: Silymarin Accelerates Liver Regeneration after Partial Hepatectomy

**DOI:** 10.1155/2017/2575273

**Published:** 2017-11-23

**Authors:** Evidence-Based Complementary and Alternative Medicine

Evidence-Based Complementary and Alternative Medicine has retracted the article titled “Silymarin Accelerates Liver Regeneration after Partial Hepatectomy” [1]. The article was found to contain images with the following concerns: There are irregularities in the background in Figures 1(a) and 1(b); the tubulin controls in Figures 1(a) and 1(b) are the same; Figure 3(b), Cyclin B (72 hrs) is the same as Figure 6(b), TGF beta1 (24 hrs); Figure 5(c), the lanes in the TGF-alpha rows at 6 hours and 72 hours do not align. The authors provided corrected figures, which are available as Supplementary Materials, but could not provide the underlying blots.
